# Computational and Mathematical Methods to Estimate the Basic Reproduction Number and Final Size for Single-Stage and Multistage Progression Disease Models for Zika with Preventative Measures

**DOI:** 10.1155/2017/4290825

**Published:** 2017-08-15

**Authors:** P. Padmanabhan, P. Seshaiyer

**Affiliations:** ^1^Foxcroft School, Middleburg, VA, USA; ^2^Department of Mathematical Sciences, George Mason University, Fairfax, VA 22030, USA

## Abstract

We present new mathematical models that include the impact of using selected preventative measures such as insecticide treated nets (ITN) in controlling or ameliorating the spread of the Zika virus. For these models, we derive the basic reproduction number and sharp estimates for the final size relation. We first present a single-stage model which is later extended to a new multistage model for Zika that incorporates more realistic incubation stages for both the humans and vectors. For each of these models, we derive a basic reproduction number and a final size relation estimate. We observe that the basic reproduction number for the multistage model converges to expected values for a standard Zika epidemic model with fixed incubation periods in both hosts and vectors. Finally, we also perform several computational experiments to validate the theoretical results obtained in this work and study the influence of various parameters on the models.

## 1. Introduction

Every year over one billion people are infected from vector-borne diseases including malaria, Dengue, chikungunya, schistosomiasis, leishmaniasis, Chagas disease, Zika, and many more. These diseases affect urban as well as rural communities but thrive primarily among communities with poor living conditions. These vector-borne diseases also impose a substantial economic burden on families and governments. Hence understanding the spread of vector-borne diseases has been a major priority for many countries.

Over the years, many factors have contributed to the increase in the spread of vector-borne diseases including the ability of the vectors to adapt to new habitats, ability of the vectors to become drug-resistant, rapid human movement, and changes in policies on control measures. Mathematical models have often been employed to quantify such dynamics of the vector-borne diseases. These models are often described as compartmental model with the populations under study divided into compartments and, with appropriate assumptions, the different subpopulations transfer between these compartments. One of the earliest models was the formulation of a simple SIR model to describe the epidemic [1–3], where the entire population being studied was divided into a susceptible class *S*(*t*) which consisted of the number of individuals who are susceptible to the disease and are not infected at time *t*, an infected class *I*(*t*) which consists of infected individuals who are assumed to be infectious and are able to spread the disease by direct contact with susceptibles, and *R*(*t*) which denotes the number of infected individuals who have been recovered and cannot spread the disease again. Most vector-borne diseases include an exposed phase *E*(*t*) between being infected and becoming infective. For a vector-borne disease this would mean a SEIR model for humans interacting with an SEI model for vectors as the vectors are not expected to recover in these models in the time span these models are solved. Currently, most models in the literature employ a SEIR-SEI single-stage model where the incubation periods are assumed to be fixed for both humans and vectors. Relaxing this assumption one may also employ a stage-progression model or the so-called linear chain trick that has been used for modeling diseases like HIV and Dengue [4–7] where the incubation may be modeled as the progression of multiple substages for humans and vectors. While these models have been considered for chikungunya and Dengue ([8, 9] and references therein), they have not been extended to Zika that also includes direct transmission until recently [10, 11]. The single-stage model introduced in [10] was extended by [11] to include symptomatic and asymptomatic infectious stages for the human population along with effect of preventative measures.

Two mathematical quantities that are often of interest in these compartmental models is the* basic reproduction number* and the* final size* [12]. The* basic reproduction number* denoted by *ℛ*_0_ is defined as the number of secondary disease cases caused by introducing a single infective individual into a wholly susceptible population of both hosts (humans) and vectors (mosquitoes). Typically if *ℛ*_0_ > 1 an epidemic occurs while if *ℛ*_0_ < 1, there will likely be no outbreak. The value of *ℛ*_0_ helps to quantify the level of control intervention necessary to contain an outbreak. For example, in the case of malaria, a mathematical model was introduced in order to show that malaria can be greatly reduced by reducing the mosquito population density below a certain threshold [13]. There are multiple ways to mathematically estimate the reproduction number for vector-borne diseases [12]. However these estimates vary considerably that may be because of different external factors such as severity of disease, the level of public health surveillance, and local climate condition that can possibly affect the number of vectors and many other such external factors [14].

The* final size* of the epidemic refers to the number of members of the population who are infected over the course of the epidemic. While there are various approaches to obtain the basic reproduction number corresponding to the model being analyzed, there are no exact solutions for obtaining final size relations for vector-borne diseases. Recently, a final size relation for epidemic models of vector-borne diseases (that also included direct transmission) was obtained for an age of infection model that can be applied to Zika [14]. Specifically, this work derived an upper and lower bound for the final size relation. This model was formulated and analyzed considering infectivity depending on age of infection which allowed arbitrary periods of stay in each compartment and also the inclusion of control measures such as treatment, quarantine, or isolation. While this work provides a new insight to understanding the epidemics of vector-transmitted diseases through a final size estimate, the authors are not aware of any other work that establishes similar estimates with an upper bound and lower bound for a traditional SEIR-SEI vector-borne disease model that includes direct transmission.

For most vector-borne diseases such as Zika, there are currently no vaccines available and resistance to drugs is an increasing threat. Hence the CDC and WHO have recommended vector control as one of the essential ways to prevent disease outbreaks. One such intervention that has shown a lot of promise in vector-borne diseases such as malaria includes using insecticide treated bednets (ITN) which has been proved to be simple, efficient, and cost-effective [15–17]. Using ITN can help reduce contacts between mosquitoes and humans at home by providing a physical barrier. The insecticide used to treat the bed net also repels mosquitoes (“excito-repellency”or “deterrence”) thus increasing the personal protection offered by the net [18, 19]. Finally, mosquitoes which are not repelled will most probably be killed as they come in contact with the insecticide as they often rest on the bed net after biting. For Zika there is a need to develop mathematical models that can help provide insight into the relation between increased coverage of ITN and the decrease of disease prevalence through a combination of the personal protection given by the repellency of the insecticide and the community protection given by its insecticidal action.

In this paper, we present the following new contributions for enhancing our understanding of the spread of Zika. First, we build on a single-stage model similar to what is considered in [10] and generalize them by including the impact of using selected preventative measures such as ITN in controlling or ameliorating the spread of the Zika virus. For this model, we derive the basic reproduction number and a sharp estimate for the final size relation. The derivation for the latter is a new alternative to the derivation of the age of infection epidemic model [14]. Specifically, we show that our result matches well with the results presented in [10, 14] in the absence of any control measures. Next, we expand the single-stage model to a new multistage model for Zika that incorporates more realistic incubation stages for both the humans and vectors. For this model also we derive a basic reproduction number and a final size relation estimate for the first time. We observe that the basic reproduction number for the multistage model converges to expected values for a standard Zika epidemic model with fixed incubation periods in both hosts and vectors. This is because both the single-stage and the multistage models would be included in an age of infection model. The proof for the final size of the multistage model builds on the derivation for the single-stage model developed in the work and the result applies also to diseases that can be transmitted directly as well as through a vector. The work in this paper incorporates the multistage progression in the intrinsic incubation periods and can be extended to include extrinsic incubation periods as well. Finally, we also perform several computational experiments to validate the theoretical results obtained in this work and study the influence of various parameters in the model.

The outline of the paper is as follows. In Section 2, we present the mathematical framework used to study the transmission dynamics and control of the Zika virus during a single outbreak via a single-stage model. We derive the basic reproduction number and a new upper and lower bound estimate for the final size relation for the single-stage model that incorporates preventative measures such as insecticide treated bed nets.  Section 3 carries out the basic analysis for an expanded multistage progression model that incorporates more incubation stages for the humans and the vectors. Finally, we present numerical results in Section 4 for both the single-stage and multistage progression models considered in the paper. Discussions and future work are presented in Section 5.

## 2. A Single-Stage Zika Model

In this work we develop and analyze an epidemic model for the spread of Zika through both a vector transmission and direct transmission via sexual contact. We will consider a constant total human population size *N*_*h*_ with *S*_*h*_(*t*) susceptibles, *E*_*h*_(*t*) exposed, *I*_*h*_(*t*) infected, and *R*_*h*_(*t*) recovered.

Let the rate of Zika transmission through biting from mosquito to human be given in terms of the biting rate *b* which corresponds to the number of bites in unit time. The effective mosquito bites in unit time that a susceptible human receives may be defined as the product of the biting rate *b* and the probability *β*_*mh*_ that a bite transmits the infection which may also be referred to as infectiousness of mosquitoes to humans. Of this a fraction *I*_*m*_/*N*_*m*_ is with an infective mosquito from *I*_*m*_(*t*). Thus the number of new infective humans in unit time is *bβ*_*mh*_*S*_*h*_(*I*_*m*_/*N*_*m*_). Defining the contact rate to be *b*_*mh*_ = *bβ*_*mh*_/*N*_*m*_, the number of new infective humans in unit time is *b*_*mh*_*S*_*h*_*I*_*m*_. The rate of the spread of Zika through direct sexual transmission from the infected human subpopulation to the susceptible human population is given by *b*_*h*_ = *a*_*h*_/*N*_*h*_ where *a*_*h*_ is the sexual transmission rate of Zika. This adds to the number of new infective humans to be *b*_*h*_*S*_*h*_*I*_*h*_.

The vectors are assumed to move from the susceptible class *S*_*m*_(*t*) to the exposed class *E*_*m*_(*t*) through biting of an infected human. For the vectors, we consider a constant birth rate contribution *μ*_*m*_*N*_*m*_ of vectors in unit time and a proportional vector death rate *μ*_*m*_ in each of the susceptible *S*_*m*_(*t*), exposed *E*_*m*_(*t*), and infected *I*_*m*_(*t*) vector classes, so that the total vector population size *N*_*m*_ is constant. We will also assume that the vectors do not recover from infection and therefore there is no recovered class for the vectors. The total number of contacts by vectors sufficient to transmit infection therefore is *β*_*hm*_ and the corresponding vector transmission rate from the infected human to the vector is given by *b*_*hm*_ = *bβ*_*hm*_/*N*_*h*_ where *β*_*hm*_ is the infectiousness of humans to mosquitoes. Therefore, the number of new infective vectors in unit time is *b*_*hm*_*S*_*m*_*I*_*h*_.

We assume that the members of the exposed class *E*_*h*_ move to become infectious at a human incubation rate of *ν*_*h*_ which is intrinsic human latent period. Members of the infectious human class recover with a rate of *γ*_*h*_. Also, we let vectors of the exposed class *E*_*m*_ move to become infectious *I*_*m*_ with a vector incubation rate *ν*_*m*_.

To incorporate preventative measures into the model, the effects of ITN are introduced in the rates of transmission from the susceptible human class to the exposed human class through a parameter measured as a percent *f* = 1 − ITN. Note that when ITN = 1 (or *f* = 0), the only movement from susceptible human class to the exposed class is through sexual transmission and not through the vector. On the other hand, if ITN = 0 (or *f* = 1), the nets have no effect and the disease can spread through both vector and sexual transmission. As in the human model, we also incorporate preventative measure ITN into the vector model. We also introduce parameter for the removal of mosquitoes denoted by *h* associated with ITN. To account for a wide range of behaviors, one can let the values of ITN from 0 to 1.

This leads to the following SEIR/SEI model for Zika transmission:

(1) Sh˙=−fbmhShIm−bhIhSh,

(2) Eh˙=fbmhShIm+bhIhSh−νhEh,

(3) Ih˙=νhEh−γhIh,

(4) Rh˙=γhIh,

(5) Sm˙=μmNm−μmSm−fbhmSmIh−pSm,

(6) Em˙=−νmEm−μmEm+fbhmSmIh−pEm,

(7) Im˙=νmEm−μmIm−pIm,

where *p* = *h* · ITN. Note that the total human population *N*_*h*_ appears implicitly in the parameters and there are no new births or deaths in the human population. Moreover, we will assume that the Zika epidemic is started by a visitor from outside the vector population *N*_*m*_. Hence we will assume *S*_*m*_(0) = *N*_*m*_ and the total population of the mosquitoes and *E*_*m*_(0) = *I*_*m*_(0) = 0.

### 2.1. Derivation of the Basic Reproduction Number

Recall that the* basic reproduction number* is defined as the number of secondary disease cases caused by introducing a single infective into a wholly susceptible population of both hosts (humans) and vectors (mosquitoes). Since the proposed mathematical model for human-vector interaction includes subpopulations with different susceptibility to infection, we will employ a general approach called the* Next Generation Matrix approach* [23–25] to find the basic reproduction number *ℛ*_0_ which is given by the following theorem.

**Theorem 1 .**

The basic reproduction number *ℛ*_0_ is given by

(8) $$\begin{array}{c} \begin{array}{c} R_{0}=\frac{1}{2}\left[\frac{a_{h}}{\gamma_{h}}+\sqrt{{\left(\frac{a_{h}}{\gamma_{h}}\right)}^{2}+4\frac{f^{2}b^{2}\beta_{hm}\beta_{mh}\nu_{m}}{\gamma_{h}\left(\mu_{m}+p\right)\left(\mu_{m}+\nu_{m}+p\right)}}\right]. \end{array} \end{array}$$

**Proof**

Given the infectious stages *E*_*h*_, *E*_*m*_, *I*_*h*_, *I*_*m*_ in (1)–(7), we can create a vector *ℱ* that represents the new infections flowing only into the exposed compartments. The components of the vector *ℱ* are obtained by considering the terms denoting new infections from the susceptible equations (1) and (5) entering the exposed equations (2) and (6) with *S*_*h*_ = *N*_*h*_ and *S*_*m*_ = N_*m*_.

(9) $$\begin{array}{c} F=\left\{\;fb_{mh}I_{m}N_{h}+a_{h}I_{h},fb_{hm}N_{m}I_{h},0,0\;\right\}. \end{array}$$

Along with *ℱ*, we will also consider *𝒱* which denote the outflow from the infectious compartments in (1)–(7) which is given by

(10) V=νhEh,νm+μm+pEm,γhIh−νhEh,−νmEm+μm+pIm.

Next, we compute the Jacobian *F* from *ℱ* given by

(11) $$\begin{array}{c} F=\left(\begin{array}{cccc} 0 & 0 & a_{h} & fb_{mh}N_{h}\\ 0 & 0 & fb_{hm}N_{m} & 0\\ 0 & 0 & 0 & 0\\ 0 & 0 & 0 & 0 \end{array}\right) \end{array}$$

and the Jacobian *V* from *𝒱* given by

(12) $$\begin{array}{c} V=\left(\begin{array}{cccc} \nu_{h} & 0 & 0 & 0\\ 0 & \nu_{m}+\mu_{m}+p & 0 & 0\\ -\nu_{h} & 0 & \gamma_{h} & 0\\ 0 & -\nu_{m} & 0 & \mu_{m}+p \end{array}\right). \end{array}$$

Using matrices *F* and *V* one can then compute the Next Generation Matrix *FV*^−1^ given by

(13) $$\begin{array}{c} {FV}^{-1}=\left(\begin{array}{cccc} \frac{a_{h}}{\gamma_{h}} & \frac{fb_{mh}N_{h}\nu_{m}}{\left(\mu_{m}+p\right)\left(\mu_{m}+\nu_{m}+p\right)} & \frac{a_{h}}{\gamma_{h}} & \frac{fb_{mh}N_{h}}{\mu_{m}}\\ \frac{fb_{hm}N_{m}}{\gamma_{h}} & 0 & \frac{fb_{hm}N_{m}}{\gamma_{h}} & 0\\ 0 & 0 & 0 & 0\\ 0 & 0 & 0 & 0 \end{array}\right). \end{array}$$

Note that (*i*, *j*) entry of the Next Generation Matrix *FV*^−1^ is the expected number of secondary infections in compartment *i* produced by individuals initially in compartment *j* assuming that the environment seen by the individual remains homogeneous for the duration of its infection. Also, matrix *FV*^−1^ is nonnegative and therefore has a nonnegative eigenvalue. The basic reproduction number can then be computed as *ℛ*_0_ = *ρ*(*FV*^−1^) which is the spectral radius of the matrix. This eigenvalue is associated with a nonnegative eigenvector which represent the distribution of infected individuals that produces the greatest number *ℛ*_0_ of secondary infections per generation. In order to calculate the eigenvalues of *FV*^−1^, we consider the characteristic equation

(14) $$\begin{array}{c} \det\left(\;FV^{-1}-\lambda I\;\right)=0, \end{array}$$

where *λ* denotes the eigenvalues of the matrix and *I* represents the Identity matrix. This can be simplified to yield

(15) $$\begin{array}{c} -\lambda^{2}\left|\begin{array}{cc} \frac{a_{h}}{\gamma_{h}}-\lambda & \frac{fb_{mh}N_{h}\nu_{m}}{\left(\mu_{m}+p\right)\left(\mu_{m}+\nu_{m}+p\right)}\\ \frac{fb_{hm}N_{m}}{\gamma_{h}} & -\lambda \end{array}\right|=0. \end{array}$$

The characteristic polynomial therefore is the following quadratic equation given by

(16) $$\begin{array}{c} \lambda^{2}-\frac{a_{h}}{\gamma_{h}}\lambda -\frac{f^{2}b_{hm}b_{mh}N_{h}N_{m}\nu_{m}}{\left(\mu_{m}+p\right)\left(\mu_{m}+\nu_{m}+p\right)\gamma_{h}}=0. \end{array}$$

The basic reproduction number *ℛ*_0_ corresponds to the dominant eigenvalue given by the root of the quadratic equation

(17) $$\begin{array}{c} \begin{array}{c} R_{0}=\frac{1}{2}\left[\frac{a_{h}}{\gamma_{h}}+\sqrt{{\left(\frac{a_{h}}{\gamma_{h}}\right)}^{2}+4\frac{f^{2}b^{2}\beta_{hm}\beta_{mh}\nu_{m}}{\gamma_{h}\left(\mu_{m}+p\right)\left(\mu_{m}+\nu_{m}+p\right)}}\right]. \end{array} \end{array}$$

**Remark 2 .**

Note that the infected human infects mosquitoes at a rate of *fb*_*hm*_*N*_*h*_/*N*_*m*_ over an average time 1/*γ*_*h*_ which produces *fb*_*hm*_*N*_*h*_/*γ*_*h*_*N*_*m*_ infected mosquitoes. Now a fraction *ν*_*m*_/(*ν*_*m*_ + *μ*_*m*_ + *p*) proceeds to become infectious. Next, the infected vectors infect humans at a rate of *fb*_*mh*_*N*_*m*_/*N*_*h*_ for an average time of 1/(*μ*_*m*_ + *p*), producing *fb*_*mh*_*N*_*m*_/*N*_*h*_(*μ*_*m*_ + *p*) infected humans per vector. The result is

(18) $$\begin{array}{c} \left[\;\frac{fb_{hm}N_{h}}{\gamma_{h}N_{m}}\;\right]\left[\;\frac{\nu_{m}}{\nu_{m}+\mu_{m}+p}\;\right]\left[\;\frac{fb_{mh}N_{m}}{N_{h}\left(\mu_{m}+p\right)}\;\right]=R_{V}. \end{array}$$

Also, sexual transmission produces *a*_*h*_ cases in average time 1/*γ*_*h*_ which then yields the additional reproductive number

(19) $$\begin{array}{c} R_{S}=\frac{a_{h}}{\gamma_{h}}. \end{array}$$

The basic reproduction number *ℛ*_0_ for system (1)–(7) can be written in terms of the basic reproduction numbers corresponding to the vector transmission *ℛ*_*V*_ and direct transmission *ℛ*_*S*_ as

(20) $$\begin{array}{c} R_{0}=\frac{1}{2}\left[\;R_{S}+\sqrt{R_{S}^{2}+4R_{V}}\;\right]. \end{array}$$

Note that Theorem 1 yields a general result for the basic reproduction number *ℛ*_0_ corresponding to the human-vector model given by equations (1)–(7) that include both sexual transmission and vector transmission. In the absence of one of these, the derived *ℛ*_0_ simplifies to physically meaningful mathematical quantities which are given in the next two corollaries.

**Corollary 3 .**

In the absence of sexual transmission (*a*_*h*_ = 0), the basic reproduction number *ℛ*_0_ only corresponds to the vector transmission, that is,

(21) $$\begin{array}{c} R_{0}=\sqrt{R_{V}}=\sqrt{\frac{f^{2}b^{2}\beta_{hm}\beta_{mh}\nu_{m}}{\gamma_{h}\left(\mu_{m}+p\right)\left(\mu_{m}+\nu_{m}+p\right)}}. \end{array}$$

**Corollary 4 .**

In the absence of vector transmission (*b* = 0), the basic reproduction number *ℛ*_0_ only corresponds to the direct (sexual) transmission given by

(22) $$\begin{array}{c} R_{0}=R_{S}=\frac{a_{h}}{\gamma_{h}}. \end{array}$$

Note that in Corollary 3 (in the absence of sexual transmission), the next generation approach employed yields a square root in the reproduction number because it views the transition from humans to vector to humans as two generations.

### 2.2. Final Size for the Single-Stage Model

In this section we will derive a relation between the* basic reproduction number* corresponding to the model equations (1)–(7) and the size of the epidemic. Note that the* final size* of the epidemic, the number of members of the population who are infected over the course of the epidemic, is *N* − *S*_*h*_(*∞*) which is often described in terms of the attack rate (1 − *S*_*h*_(*∞*)/*N*_*h*_). We will first prove a lemma that will be used to derive the final size relation.

**Lemma 5 .**

For system (1)–(7), the total number of infected vectors depends on the dynamics of the epidemic and the total number of human infections as follows:

(23) $$\begin{array}{c} \overset{\infty }{\underset{0}{\int }}I_{m}dt=\frac{fb_{hm}\nu_{m}S_{m}^{*}}{\left(\mu_{m}+p\right)\left(\nu_{m}+\mu_{m}+p\right)}\overset{\infty }{\underset{0}{\int }}I_{h}dt, \end{array}$$

where min⁡*S*_*m*_ ≤ *S*_*m*_^*∗*^ ≤ max⁡*S*_*m*_ ≤ *N*_*m*_.

**Proof**

Integrating (7), we get

(24) $$\begin{array}{c} I_{m}\left(\;\infty \;\right)-I_{m}\left(\;0\;\right)=\overset{\infty }{\underset{0}{\int }}\nu_{m}E_{m}dt-\overset{\infty }{\underset{0}{\int }}\left(\;\mu_{m}+p\;\right)I_{m}dt. \end{array}$$

Letting *I*_*m*_(*∞*) − *I*_*m*_(0) = 0, we have

(25) $$\begin{array}{c} \overset{\infty }{\underset{0}{\int }}I_{m}dt=\frac{\nu_{m}}{\mu_{m}+p}\overset{\infty }{\underset{0}{\int }}E_{m}dt. \end{array}$$

Now integrating (6), we get

(26) Em∞−Em0=−∫0∞νm+μm+pEmdt+∫0∞fbhmSmIhdt.

Again, noting that *E*_*m*_(*∞*) = *E*_*m*_(0) = 0, this reduces to

(27) $$\begin{array}{c} \overset{\infty }{\underset{0}{\int }}E_{m}dt=\frac{fb_{hm}}{\nu_{m}+\mu_{m}+p}\overset{\infty }{\underset{0}{\int }}S_{m}I_{h}dt. \end{array}$$

Since *I*_*h*_ ≥ 0 on the interval [0, *∞*), one can use the integral mean value theorem and we have

(28) $$\begin{array}{c} \overset{\infty }{\underset{0}{\int }}S_{m}I_{h}dt=S_{m}^{*}\overset{\infty }{\underset{0}{\int }}I_{h}dt, \end{array}$$

where min⁡*S*_*m*_ ≤ *S*_*m*_^*∗*^ ≤ max⁡*S*_*m*_ ≤ *N*_*m*_. Substituting (28) into (27), we have

(29) $$\begin{array}{c} \overset{\infty }{\underset{0}{\int }}E_{m}dt=\frac{fb_{hm}S_{m}^{*}}{\nu_{m}+\mu_{m}+p}\overset{\infty }{\underset{0}{\int }}I_{h}dt. \end{array}$$

Finally, substituting (29) in (25) completes the proof of the lemma.

Next, we prove the following theorem that provides an upper bound for the final size for system (1)–(7) in terms of a basic reproduction number ${\hat{ℛ}}_{0}$ that is closely related to *ℛ*_0_ that was derived in the previous section.

**Theorem 6 .**

For equations (1)–(7) the final size relation can be bounded above as follows:

(30) $$\begin{array}{c} \log\frac{S_{h}\left(0\right)}{S_{h}\left(\infty \right)}\leq {\hat{R}}_{0}\left[\;1-\frac{S_{h}\left(\infty \right)}{N_{h}}\;\right], \end{array}$$

where the basic reproduction number ${\hat{ℛ}}_{0}$ is the sum of the sexual transmission reproduction number *ℛ*_*S*_ and the vector transmission reproduction number *ℛ*_*V*_ given by ${\hat{ℛ}}_{0}={ℛ}_{S}+{ℛ}_{V}$.

**Proof**

Adding equations (1)–(3) yields the following:

(31) $$\begin{array}{c} {\left(\;S_{h}\left(\;t\;\right)+E_{h}\left(\;t\;\right)+I_{h}\left(\;t\;\right)\;\right)}^{\prime }=-\gamma_{h}I_{h}. \end{array}$$

This implies that *S*_*h*_(*t*) + *E*_*h*_(*t*) + *I*_*h*_(*t*) is a positive decreasing function and therefore the limit exists. The derivative of positive decreasing function tends to zero, and this yields that −*γ*_*h*_*I*_*h*_ → 0 and since *γ*_*h*_ > 0, this implies that *I*_*h*_ → 0.

Integrating (31) we get

(32) $$\begin{array}{c} {\left.\;\left(\;S_{h}\left(\;t\;\right)+E_{h}\left(\;t\;\right)+I_{h}\left(\;t\;\right)\;\right)\;\right|}_{0}^{\infty }=-\gamma_{h}\overset{\infty }{\underset{0}{\int }}I_{h}dt. \end{array}$$

Noting that *E*_*h*_(*∞*) = *I*_*h*_(*∞*) = 0 and *S*_*h*_(0) + *E*_*h*_(0) + *I*_*h*_(0) = *N*_*h*_, (32) simplifies to the following:

(33) $$\begin{array}{c} \gamma_{h}\overset{\infty }{\underset{0}{\int }}I_{h}dt=N_{h}-S_{h}\left(\;\infty \;\right). \end{array}$$

Employing the technique of Separation of Variables on (1):

(34) $$\begin{array}{c} \overset{\infty }{\underset{0}{\int }}\frac{dS_{h}}{S_{h}}dt=-fb_{mh}\overset{\infty }{\underset{0}{\int }}I_{m}dt-b_{h}\overset{\infty }{\underset{0}{\int }}I_{h}dt. \end{array}$$

Substituting (33) and simplifying yields

(35) $$\begin{array}{c} \overset{\infty }{\underset{0}{\int }}\frac{dS_{h}}{S_{h}}dt=-fb_{mh}\overset{\infty }{\underset{0}{\int }}I_{m}dt-b_{h}\left[\;\frac{N_{h}-S_{h}\left(\infty \right)}{\gamma_{h}}\;\right]. \end{array}$$

Integrating the left hand side and using the definition of the rate of sexual transmission *a*_*h*_ = *b*_*h*_*N*_*h*_ we get

(36) $$\begin{array}{c} \log\frac{S_{h}\left(0\right)}{S_{h}\left(\infty \right)}=\frac{a_{h}}{\gamma_{h}}\left[\;1-\frac{S_{h}\left(\infty \right)}{N_{h}}\;\right]+fb_{mh}\overset{\infty }{\underset{0}{\int }}I_{m}dt. \end{array}$$

Using Lemma 5, we can substitute (23) into (36) to yield

(37) log⁡Sh0Sh∞=ahγh1−Sh∞Nh+f2bhmbmhνmSm∗μm+pνm+μm+p∫0∞Ihdt,

where min⁡*S*_*m*_ ≤ *S*_*m*_^*∗*^ ≤ max⁡*S*_*m*_ ≤ *N*_*m*_. Using (33) this reduces to

(38) log⁡Sh0Sh∞=ahγh+f2b2βhmβmhνmSm∗γhμm+pμm+νm+pNm·1−Sh∞Nh=RS+Sm∗NmRV1−Sh∞Nh≤RS+RV1−Sh∞Nh=R^01−Sh∞Nh,

where we have used the fact that *S*_*m*_^*∗*^ ≤ *N*_*m*_.

**Remark 7 .**

Note that integrating (1) and adding (1), (2), and (3) from 0 to *t*, we get

(39) log⁡Sh0Shtbmh∫0∞Imtdt+bh∫0∞Ihtdt=R^0NhNh−Sht−Eht−Iht.

This leads to the form

(40) Sht+Eht+Iht−NhR^0log⁡Sht=Nh−NhR^0log⁡Sh0.

One can use this implicit relation between *S*_*h*_(*t*), *E*_*h*_(*t*), and *I*_*h*_(*t*) to describe the orbit of solutions. In addition since the right hand side of (30) is finite, the left hand side is also finite and this shows *S*_*h*_(*∞*) > 0.

Next, we prove an estimate that provides a lower bound for the final size relation. The proof relies on the assumption that the vector population has a much faster time scale than the host population and therefore the vector population is at a quasi-steady-stage equilibrium, given by solutions to the equations for *S*_*m*_, *E*_*m*_, and *I*_*m*_ in (1)–(7) that are constant functions of *t*, but may depend on *S*_*h*_(*t*), *E*_*h*_(*t*), and *I*_*h*_(*t*).

**Theorem 8 .**

Let the vector population be at a quasi-steady-stage equilibrium. The final size relation for (1)–(7) can then be bounded below as follows:

(41) $$\begin{array}{c} \log\frac{S_{h}\left(0\right)}{S_{h}\left(\infty \right)}\geq R_{0}^{*}\left[\;1-\frac{S_{h}\left(\infty \right)}{N_{h}}\;\right], \end{array}$$

where *ℛ*_0_^*∗*^ is given by

(42) $$\begin{array}{c} R_{0}^{*}=\left[\;R_{S}+\frac{\mu_{m}}{\mu_{m}+p+fb\beta_{hm}}R_{V}\;\right]. \end{array}$$

**Proof**

To determine the lower bound for log⁡(*S*_*h*_(0)/*S*_*h*_(*∞*)), we will first obtain a minimum for the Susceptible vector population *S*_*m*_. Since the vector population is assumed to be at a quasi-steady-stage equilibrium, we let $\dot{S_{m}}(t)=0$ in (5) which yields

(43) $$\begin{array}{c} \mu_{m}N_{m}-\left(\;\mu_{m}+p\;\right)S_{m}-fb_{hm}S_{m}I_{h}=0. \end{array}$$

This can be rewritten to give

(44) $$\begin{array}{c} S_{m}=\frac{\mu_{m}N_{m}}{\mu_{m}+p+fb_{hm}I_{h}}\geq \frac{\mu_{m}N_{m}}{\mu_{m}+p+fb\beta_{hm}} \end{array}$$

since *b*_*hm*_ = *bβ*_*hm*_/*N*_*h*_ and *I*_*h*_ ≤ *N*_*h*_. Therefore,

(45) log⁡Sh0Sh∞=RS+Sm∗NmRV1−Sh∞Nh≥RS+μmμm+p+fbβhmRV1−Sh∞Nh=R0∗1−Sh∞Nh.

**Remark 9 .**

Theorems 6 and 8 provide an upper and lower bound estimate for the final size relation, respectively, to yield

(46) R0∗1−Sh∞Nhlog⁡Sh0Sh∞≤R^01−Sh∞Nh.

Note that for *f* = 1 and *p* = 0, we are able to recover similar estimates that were derived for an age of infection epidemic model that included both vector transmission and direct (sexual) transmission [14].

To summarize, we have introduced the following variations of basic reproduction number in the description for the single-stage model:

(47) R0=Basic  Reproduction  Number,RS=Basic  Reproduction  Number  for  Direct  Transmission,RV=Basic  Reproduction  Number  for  Vector  Transmission,R^0=RS+RV,R0∗=RS+μmμm+p+fbβhmRV.

**Remark 10 .**

Note that in the derivation of the final size for the single-stage model we have assumed that *I*_*m*_(*∞*) − *I*_*m*_(0) = 0. In general, however, one can expect *I*_*m*_(*∞*) − *I*_*m*_(0) = *C* < 0 (as we expect the infections to die out). In this case, one can prove the upper bound estimate (30) as before; however, the lower bound estimate (41) is not a sharp estimate. This is not considered in this paper.

## 3. A Multistage Progression Zika Model

In this section we extend the single-stage Zika epidemic model to a multistage model by incorporating more realistic incubation period distributions. Specifically, we relax the assumption of fixed incubation period by using a stage-progression model or the so-called linear chain trick [4–7]. This Zika model incorporates incubation periods as the progression in *e*_*h*_ incubation substages in humans (*E*_*h*_1__, *E*_*h*_2__,…, *E*_*h*_*e*_*h*___) and *e*_*m*_ incubation substages in vectors (*E*_*m*_1__, *E*_*m*_2__,…, *E*_*m*_*e*_*m*___). This idea of a stage-progression model has been used for Dengue [7] which is caused by the same species of mosquito that causes Zika. The reason for introducing incubation substages is to model (for the first time for Zika) the time between a human is infected and the onset of symptoms due to the infection. These periods are important determinants of the temporal dynamics of the ZIKV transmission and are therefore critical for clinical diagnosis, outbreak investigation, implementation of prevention, programming control measures, and mathematical modeling. Under this formulation, the resulting incubation periods follow a gamma distribution with integer parameters *e*_*h*_ and *e*_*m*_, respectively. When the rates of progression between substages are given by *e*_*h*_*k*_*h*_ and *e*_*m*_*k*_*m*_ for the incubation periods, the resulting gamma distribution has means 1/*k*_*h*_ and 1/*k*_*m*_ for the incubation periods, respectively, and the corresponding variances are given by 1/*e*_*h*_*k*_*h*_^2^ and 1/*e*_*m*_*k*_*m*_^2^, respectively [7]. In this model we incorporate this stage-progression only in the intrinsic incubation period in humans and vectors. The analysis will be similar if one were to also incorporate stage-progression in the infectious period for the humans also.

We then have the following system of nonlinear differential equations describing the dynamics of Zika through a human-vector interaction as

(48) Sh˙=−fbmhShIm−bhIhSh,

(49) Eh1˙=fbmhShIm+bhIhSh−ehνhEh1,

(50) Ehj˙=ehνhEhj−1−ehνhEhj,2⩽j⩽eh,

(51) Ih˙=ehνhEheh−γhIh,

(52) Rh˙=γhIh,

(53) Sm˙=μmNm−μmSm−fbhmSmIh−pSm,

(54) Em1˙=−emνmEm1−μmEm1+fbhmSmIh−pEm1,

(55) Emj˙=emνmEmj−1−emνmEmj−μmEmj−pEmj,2⩽j⩽em,

(56) Im˙=emνmEmem−μmIm−pIm.

In this section we prove a theorem that provides estimates for the final size for the system (48)–(56). First, we will first prove a lemma that will be used to derive the final size relation.

**Lemma 11 .**

For system (48)–(56), the total number of infected vectors depends on the dynamics of the epidemic and the total number of human infections as follows:

(57) ∫0∞Imdt=fbhmμm+pemνmemνm+μm+pem∫0∞SmIhdt.

**Proof**

Integrating (56), we get

(58) Im∞−Im0=∫0∞emνmEmemdt−∫0∞μm+pImdt.

Letting *I*_*m*_(*∞*) − *I*_*m*_(0) = 0, we have

(59) $$\begin{array}{c} \overset{\infty }{\underset{0}{\int }}I_{m}dt=\frac{e_{m}\nu_{m}}{\left(\mu_{m}+p\right)}\overset{\infty }{\underset{0}{\int }}E_{m_{e_{m}}}dt. \end{array}$$

Note that, for *j* = *e*_*m*_, (55) can be written as

(60) $$\begin{array}{c} \dot{E_{m_{e_{m}}}}=e_{m}\nu_{m}E_{m_{e_{m}-1}}-e_{m}\nu_{m}E_{m_{e_{m}}}-\mu_{m}E_{m_{e_{m}}}-pE_{m_{e_{m}}}. \end{array}$$

Integrating this equation we get

(61) Emem∞−Emem0=∫0∞emνmEmem−1dt−∫0∞μm+emνm+pEmemdt.

Noting that *E*_*m*_*e*_*m*___(*∞*) = *E*_*m*_*e*_*m*___(0) = 0, this reduces to

(62) $$\begin{array}{c} \overset{\infty }{\underset{0}{\int }}E_{m_{e_{m}}}dt=\frac{e_{m}\nu_{m}}{e_{m}\nu_{m}+\mu_{m}+p}\overset{\infty }{\underset{0}{\int }}E_{m_{e_{m}-1}}dt. \end{array}$$

Substituting, (62) into (59), we get

(63) $$\begin{array}{c} \overset{\infty }{\underset{0}{\int }}I_{m}dt=\frac{{\left(e_{m}\nu_{m}\right)}^{2}}{\left(\mu_{m}+p\right)\left(e_{m}\nu_{m}+\mu_{m}+p\right)}\overset{\infty }{\underset{0}{\int }}E_{m_{e_{m}-1}}dt. \end{array}$$

Next for *j* = *e*_*m*_ − 1, (55) can be written as

(64) Emem−1˙=emνmEmem−2−emνmEmem−1−μmEmem−1−pEmem−1.

Integrating this equation we get

(65) Emem−1∞−Emem−10=∫0∞emνmEmem−2dt−∫0∞μm+emνm+pEmem−1dt.

Substituting *E*_*m*_*e*_*m*_−1__(*∞*) = *E*_*m*_*e*_*m*_−1__(0) = 0 this reduces to

(66) $$\begin{array}{c} \overset{\infty }{\underset{0}{\int }}E_{m_{e_{m}-1}}dt=\frac{e_{m}\nu_{m}}{e_{m}\nu_{m}+\mu_{m}+p}\overset{\infty }{\underset{0}{\int }}E_{m_{e_{m}-2}}dt. \end{array}$$

Substituting (66) into (63), we get

(67) ∫0∞Imdt=emνm3μm+pemνm+μm+p2∫0∞Emem−2dt.

Repeating this process inductively one can easily show that

(68) ∫0∞Imdt=emνmemμm+pemνm+μm+pem−1∫0∞Em1dt.

Integrating (54) we get

(69) Em1∞−Em10=−emνm+μm+p∫0∞Em1+fbhm∫0∞SmIhdt.

Again, noting *E*_*m*_1__(*∞*) − *E*_*m*_1__(0) = 0, this can be simplified to yield

(70) $$\begin{array}{c} \overset{\infty }{\underset{0}{\int }}E_{m_{1}}dt=\frac{fb_{hm}}{e_{m}\nu_{m}+\mu_{m}+p}\overset{\infty }{\underset{0}{\int }}S_{m}I_{h}dt. \end{array}$$

Substituting (70) into (68) finally yields

(71) ∫0∞Imdt=fbhmμm+pemνmemνm+μm+pem∫0∞SmIhdt

which completes the proof of the lemma.

**Theorem 12 .**

For (48)–(52) the final size relation satisfies the following upper bound estimate:

(72) $$\begin{array}{c} \log\frac{S_{h}\left(0\right)}{S_{h}\left(\infty \right)}\leq {\bar{R}}_{0}\left[\;1-\frac{S_{h}\left(\infty \right)}{N_{h}}\;\right], \end{array}$$

where ${\bar{ℛ}}_{0}$, the basic reproduction number corresponding to the system, is the sum of the reproduction numbers corresponding to direct (sexual) transmission *ℛ*_*S*_ and vector transmission ${\hat{ℛ}}_{V}$ which are given by

(73) RS=ahγh,R^V=f2b2βmhβhmμm+pγhemνmemνm+μm+pem.

**Proof**

Adding (48)–(51) yields the following:

(74) $$\begin{array}{c} \left(\;S_{h}\left(\;t\;\right)+\overset{e_{h}}{\underset{j=1}{\sum }}E_{h_{j}}\left(\;t\;\right)+I_{h}\left(\;t\;\right)\;\right)=-\gamma_{h}I_{h}\left(\;t\;\right). \end{array}$$

This implies that *S*_*h*_(*t*) + ∑_*j*=1_^*e*_*h*_^*E*_*h*_*j*__(*t*) + *I*_*h*_(*t*) is a positive decreasing function and therefore the limit exists. The derivative of positive decreasing function tends to zero, and this yields that −*γ*_*h*_*I*_*h*_ → 0 and since *γ*_*h*_ > 0, this implies that *I*_*h*_ → 0.

Integrating (74) we get

(75) $$\begin{array}{c} {\left.\;\left(\;S_{h}\left(\;t\;\right)+\sum_{j=1}^{e_{h}}E_{h_{j}}\left(\;t\;\right)+I_{h}\left(\;t\;\right)\;\right)\;\right|}_{0}^{\infty }=-\gamma_{h}\overset{\infty }{\underset{0}{\int }}I_{h}dt. \end{array}$$

Noting that *E*_*h*_*j*__(*∞*) = *I*_*h*_(*∞*) = 0 and *S*_*h*_(0) + ∑_*j*=1_^*e*_*h*_^*E*_*h*_*j*__(0) + *I*_*h*_(0) = *N*_*h*_, (75) simplifies to the following:

(76) $$\begin{array}{c} \gamma_{h}\overset{\infty }{\underset{0}{\int }}I_{h}dt=N_{h}-S_{h}\left(\;\infty \;\right). \end{array}$$

Employing (48):

(77) $$\begin{array}{c} \overset{\infty }{\underset{0}{\int }}\frac{dS_{h}}{S_{h}}dt=-fb_{mh}\overset{\infty }{\underset{0}{\int }}I_{m}dt-b_{h}\overset{\infty }{\underset{0}{\int }}I_{h}dt. \end{array}$$

Substituting (76) and simplifying yields

(78) $$\begin{array}{c} \overset{\infty }{\underset{0}{\int }}\frac{dS_{h}}{S_{h}}dt=-fb_{mh}\overset{\infty }{\underset{0}{\int }}I_{m}dt-b_{h}\left[\;\frac{N_{h}-S_{h}\left(\infty \right)}{\gamma_{h}}\;\right]. \end{array}$$

Integrating the left hand side and since the rate of sexual transmission *a*_*h*_ = *b*_*h*_*N*_*h*_ we get

(79) $$\begin{array}{c} \log\frac{S_{h}\left(0\right)}{S_{h}\left(\infty \right)}=\frac{a_{h}}{\gamma_{h}}\left[\;1-\frac{S_{h}\left(\infty \right)}{N_{h}}\;\right]+fb_{mh}\overset{\infty }{\underset{0}{\int }}I_{m}dt. \end{array}$$

Substituting (57) from Lemma 11 into (79) gives us

(80) log⁡Sh0Sh∞=ahγh1−Sh∞Nh+f2bmhbhmμm+pemνmemνm+μm+pem∫0∞SmIhdt≤ahγh1−Sh∞Nh+f2bmhbhmSm∗μm+pemνmemνm+μm+pem∫0∞Ihdt,

where we have used the integral mean value theorem as *I*_*h*_ ≥ 0 on the interval [0, *∞*) with min⁡*S*_*m*_ ≤ *S*_*m*_^*∗*^ ≤ max⁡*S*_*m*_ ≤ *N*_*m*_. Using (76) we can finally conclude that

(81) log⁡Sh0Sh∞=ahγh+f2bmhbhmSm∗μm+pemνmemνm+μm+pem·1−Sh∞Nh=ahγh+f2b2βmhβhmSm∗μm+pγhNmemνmemνm+μm+pem·1−Sh∞Nh≤ahγh+f2b2βmhβhmμm+pγhemνmemνm+μm+pem·1−Sh∞Nh,

where we have used the fact that *S*_*m*_^*∗*^ ≤ *N*_*m*_. Define the reproduction numbers corresponding to direct (sexual) transmission *ℛ*_*S*_ and vector transmission ${\hat{ℛ}}_{V}$, respectively, to be

(82) RS=ahγh,R^V=f2b2βmhβhmμm+pγhemνmemνm+μm+pem;

we can now get the following upper bound estimate satisfied by the final size given by

(83) $$\begin{array}{c} \log\frac{S_{h}\left(0\right)}{S_{h}\left(\infty \right)}\leq {\bar{R}}_{0}\left[\;1-\frac{S_{h}\left(\infty \right)}{N_{h}}\;\right], \end{array}$$

where ${\bar{ℛ}}_{0}={ℛ}_{S}+{\hat{ℛ}}_{V}$. This proves the upper bound.

**Corollary 13 .**

As the number of stages *e*_*m*_ → *∞*, the basic reproduction number ${\hat{ℛ}}_{V}$ corresponding to only vector transmission converges to the basic reproduction number ${\hat{ℛ}}_{V}^{*}$ corresponding to a model that incorporates infected mosquitoes experiencing a fixed incubation period 1/*ν*_*m*_ that is followed by an infectious state from which vectors do not recover. In particular,

(84) $$\begin{array}{c} {\hat{R}}_{V}^{*}=\underset{e_{m}\to \infty }{\lim}{\hat{R}}_{V}=\frac{f^{2}b^{2}\beta_{mh}\beta_{hm}}{\left(\mu_{m}+p\right)\gamma_{h}}e^{-\left(\mu_{m}+p\right)/\nu_{m}}. \end{array}$$

**Proof**

Using the definition of ${\hat{ℛ}}_{V}$ we have

(85) R^V∗=limem→∞⁡R^V=limem→∞⁡f2b2βmhβhmμm+pγhemνmemνm+μm+pem=f2b2βmhβhmμm+pγhlimem→∞⁡emνmemνm+μm+pem=f2b2βmhβhmμm+pγh1limem→∞⁡1+μm+p/emνmem=f2b2βmhβhmμm+pγh1limx→∞⁡1+1/xxμm+p/νm=f2b2βmhβhmμm+pγhe−μm+p/νm,

where *x* = *e*_*m*_*ν*_*m*_/(*μ*_*m*_ + *p*).

Next, we prove an estimate that provides a lower bound for the final size relation. As in the single-stage model, the proof relies on the assumption that the vector population has a much faster time scale than the host population and therefore the vector population is at a quasi-steady-stage equilibrium, given by solutions to (48)–(56) that are constant functions of *t*.

**Theorem 14 .**

Let the vector population be at a quasi-steady-stage equilibrium. The final size relation for equations (48)–(56) can then be bounded below as follows:

(86) $$\begin{array}{c} \log\frac{S_{h}\left(0\right)}{S_{h}\left(\infty \right)}\geq {\bar{R}}_{0}^{*}\left[\;1-\frac{S_{h}\left(\infty \right)}{N_{h}}\;\right], \end{array}$$

where ${\bar{ℛ}}_{0}^{*}$ is given by

(87) $$\begin{array}{c} {\bar{R}}_{0}^{*}=\left[\;\frac{\mu_{m}}{\mu_{m}+p+fb\beta_{hm}}{\hat{R}}_{V}+R_{S}\;\right]. \end{array}$$

**Proof**

To prove the lower bound, recall that

(88) $$\begin{array}{c} \log\frac{S_{h}\left(0\right)}{S_{h}\left(\infty \right)}=\left(\;R_{S}+\frac{S_{m}^{*}}{N_{m}}{\hat{R}}_{V}\;\right)\left[\;1-\frac{S_{h}\left(\infty \right)}{N_{h}}\;\right]. \end{array}$$

Also, since the vector population is assumed to be at a quasi-steady-stage equilibrium, setting $\dot{S_{m}}=0$ in (53) yields

(89) $$\begin{array}{c} S_{m}=\frac{\mu_{m}N_{m}}{\mu_{m}+p+fb_{hm}I_{h}}\geq \frac{\mu_{m}N_{m}}{\mu_{m}+p+fb\beta_{hm}} \end{array}$$

since *b*_*hm*_ = *bβ*_*hm*_/*N*_*h*_ and *I*_*h*_ ≤ *N*_*h*_. Substituting (89) in (88) then gives

(90) log⁡Sh0Sh∞≥RS+μmμm+p+fbβhmR¯V1−Sh∞Nh=R¯0∗1−Sh∞Nh.

**Remark 15 .**

Theorems 12 and 14 together provide an upper and lower bound estimate, respectively, for the final size relation for the model (48)–(56) given by

(91) R¯0∗1−Sh∞Nhlog⁡Sh0Sh∞≤R¯01−Sh∞Nh.

**Remark 16 .**

Note that in the derivation of the final size for the multistage model we have assumed that *I*_*m*_(*∞*) − *I*_*m*_(0) = 0. In general, however, one can expect *I*_*m*_(*∞*) − *I*_*m*_(0) = *C* < 0 (as we expect the infections to die out). In this case, one can prove the upper bound estimate (72) as before; however, the lower bound estimate (86) is not a sharp estimate. This is not considered in this paper.

To summarize, we have introduced the following variations of basic reproduction number in the description for the multistage model:

(92) RS=Basic  Reproduction  Number  for  Direct  Transmission,R^V=f2b2βmhβhmμm+pγhemνmemνm+μm+pem,R¯0=RS+R^V,R^V∗=limem→∞⁡R^V=f2b2βmhβhmμm+pγhe−μm+p/νm,R¯0∗=μmμm+p+fbβhmR^V+RS.

## 4. Computational Experiments

In this section, we validate our theoretical results developed in this work and perform simulations to predict the dynamics and estimate the basic reproduction numbers and final size relations. We implement the solution to the single-stage system (1)–(7) and the multistage system (48)–(56) in MATLAB using a fourth order Runge-Kutta method for solving ordinary differential equations. Specifically, the single-stage system of differential equations is solved using the script ode45 from MATLAB [26].

For our simulations, we considered the total human and vector populations to be *N*_*h*_ = 1000 and *N*_*m*_ = 4000, respectively, as in [14]. We considered one infective human initially and that the Zika epidemic is started by a visiting vector from outside the vector population *N*_*m*_.

For the parameters in the models, we refer mainly to the data used in [10, 14] that corresponded to the 2015 Zika outbreak in Barranquilla, Colombia. With the values for the parameters in Table 1, a linear relation was proposed in [14] given by

(93) $$\begin{array}{c} 11b_{hm}b_{mh}+6.48a_{h}=2.676. \end{array}$$

This relation restricted the value of rate of sexual transmission as 0 ≤ *a*_*h*_ ≤ 0.413. The dynamics of all the subpopulations both for the humans and the vector are computed for varying values of *a*_*h*_ and varying values of control measures ITN for both the single-stage and multistage models. For the latter, we also consider the effect of the number of incubation stages in the model dynamics.

**Figure 1:**
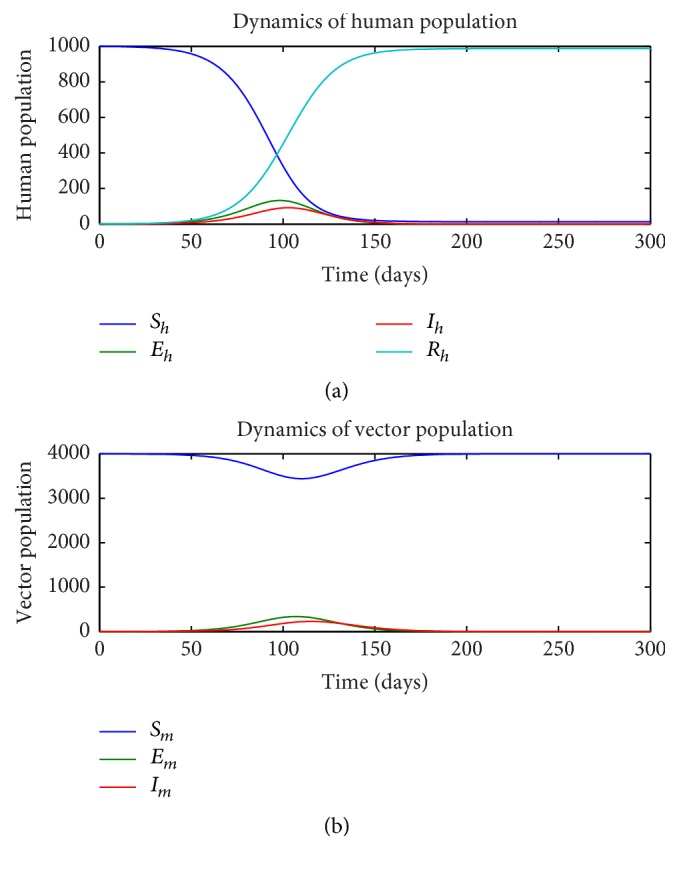
Dynamics of human (a) and vector (b) subpopulations for *a*_*h*_ = 0 and ITN = 0.

**Figure 2:**
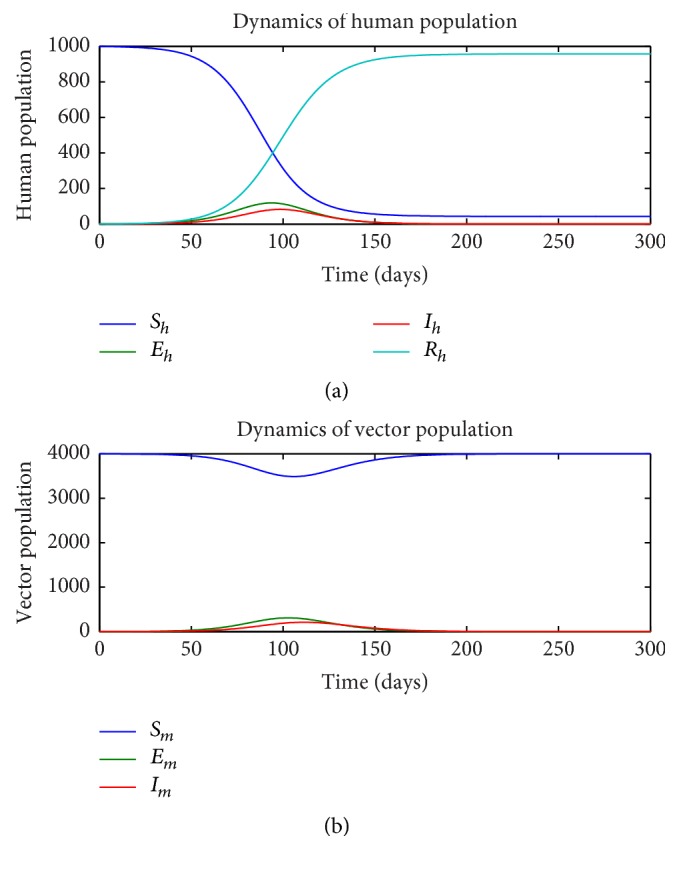
Dynamics of human (a) and vector (b) subpopulations for *a*_*h*_ = 0.2 and ITN = 0.

**Figure 3:**
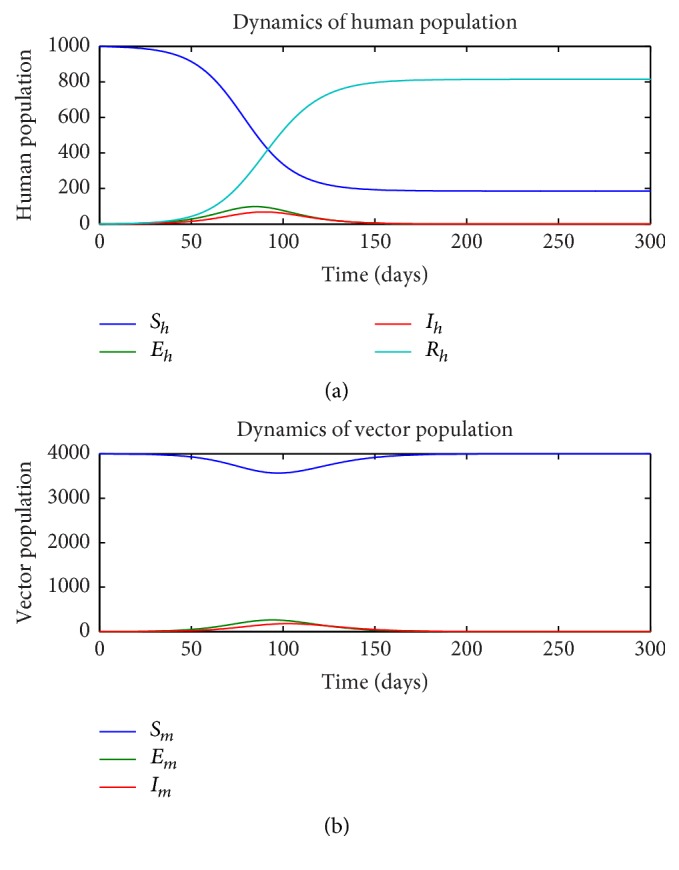
Dynamics of human (a) and vector (b) subpopulations for *a*_*h*_ = 0.413 and ITN = 0.

First, we consider the dynamics of the single-stage model (1)–(7) in the absence of insecticide treated nets (ITN = 0) and as a function of the rate of sexual transmission *a*_*h*_. Figures 1–3 illustrate the dynamics of the various states in the human (a) and vector (b) for a pure vector transmission (*a*_*h*_ = 0), a combination of both direct and vector transmission (*a*_*h*_ = 0.2), and a pure direct (sexual) transmission (*a*_*h*_ = 0.413). We note that as the value of *a*_*h*_ increases, the nature of the graphs also change. Specifically note that the final value of the number of susceptible humans *S*_*h*_(*t*) decreases as *a*_*h*_ increases.

**Figure 4:**
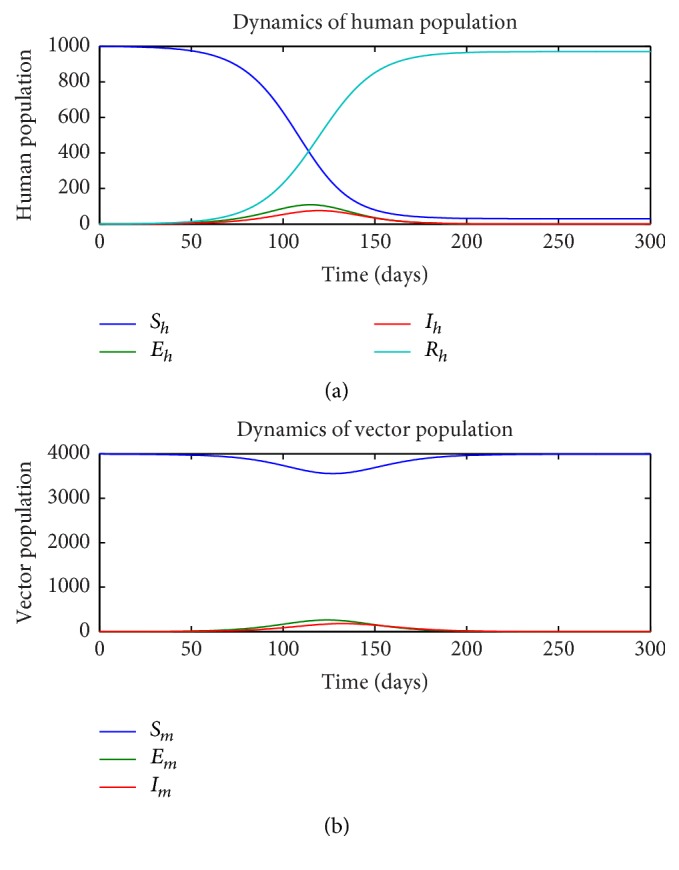
Dynamics of human (a) and vector (b) subpopulations for *a*_*h*_ = 0 and ITN = 0.1.

**Figure 5:**
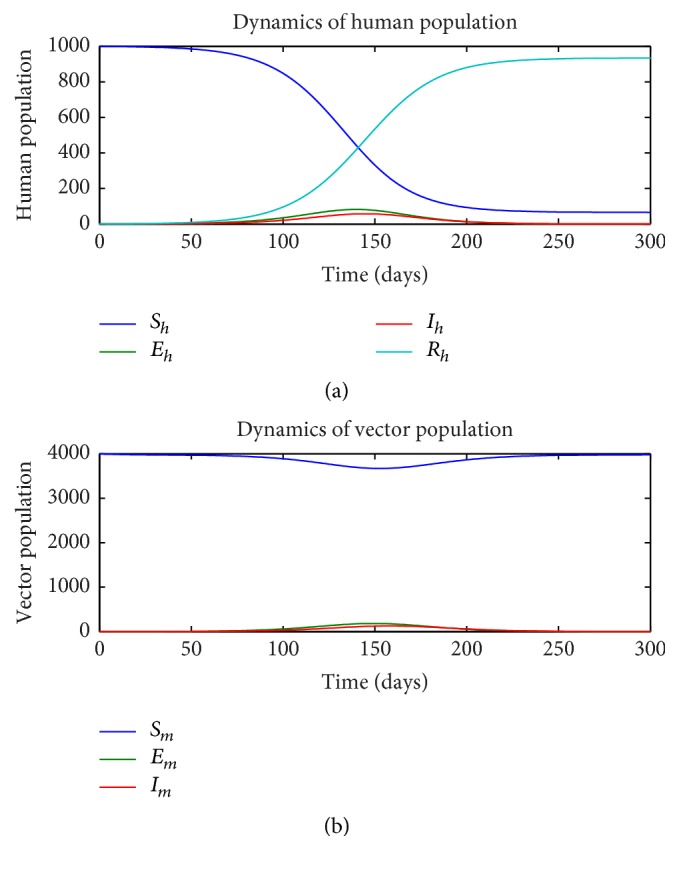
Dynamics of human (a) and vector (b) subpopulations for *a*_*h*_ = 0 and ITN = 0.2.

**Figure 6:**
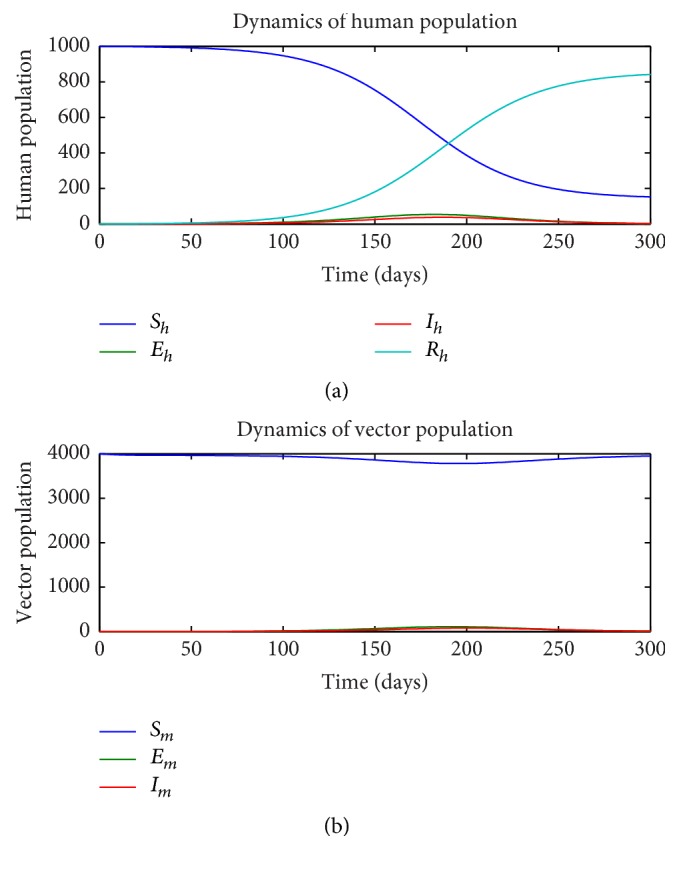
Dynamics of human (a) and vector (b) subpopulations for *a*_*h*_ = 0 and ITN = 0.3.

Next, we consider the effect of the insecticide treated nets with no sexual transmission. So we let *a*_*h*_ = 0 and let ITN = 0.1,0.2,0.3. The results are illustrated in Figures 4, 5, and 6, respectively. We clearly note that as the value of ITN increases in percentage (10%, 20%, 30%) of protection because of the nets, it takes more time for humans to get infected. Clearly this shows the effect of using nets.

**Figure 7:**
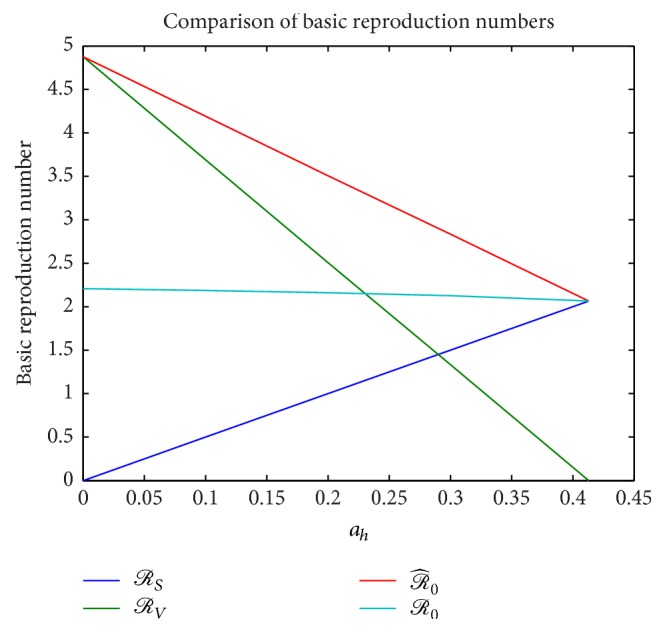
Comparison of the basic reproduction numbers for ITN = 0 and increasing values of *a*_*h*_ = 0,0.1,0.2,0, 3,0.4,0.413.

**Figure 8:**
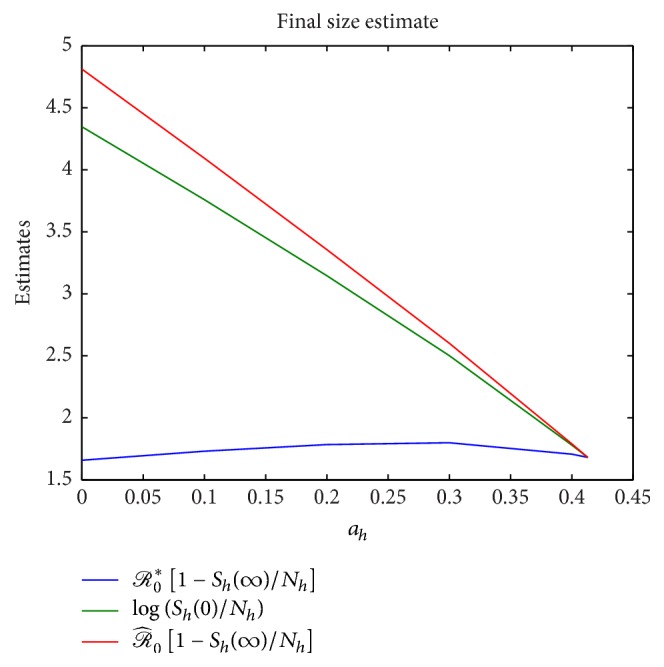
Comparison of the basic reproduction numbers for ITN = 0 and increasing values of *a*_*h*_ = 0,0.1,0.2,0, 3,0.4,0.413.

Next, we consider the influence on the rate of sexual transmission *a*_*h*_ on the various basic reproduction numbers that were obtained for the single-stage model. These include *ℛ*_0_ derived using the Next Generation Matrix approach in (20), the basic reproduction number corresponding to purely direct (sexual) transmission *ℛ*_*S*_ defined in (19), the basic reproduction number corresponding to purely vector transmission *ℛ*_*V*_ defined in (18), and the basic reproduction number ${\hat{ℛ}}_{0}$ obtained in Theorem 6 which is the sum of *ℛ*_*S*_ and *ℛ*_*V*_.  Figure 7 compares these reproduction numbers as *a*_*h*_ increases from 0 to 0.413. Next, we perform numerical experiments to validate the upper bound and lower bound estimate for the final size relation obtained in (46). This is illustrated in Figure 8 which clearly demonstrates the validity of the estimate. One may also note that log⁡(*S*_*h*_(0)/*S*_*h*_(*∞*)) is closer to the upper bound as pointed out in [14]. Also, for a purely direct (sexual) transmission, ${\hat{ℛ}}_{0}={\hat{ℛ}}_{0}^{*}$ and therefore we get equality in the estimate that is denoted by the convergence at *a*_*h*_ = 0.413 in Figure 8.

Next, we considered the sharpness of the upper bound estimate by solving for *S*_*h*_(*∞*) in

(94) $$\begin{array}{c} \log\frac{S_{h}\left(0\right)}{S_{h}\left(\infty \right)}-{\hat{R}}_{0}\left[\;1-\frac{S_{h}\left(\infty \right)}{N_{h}}\;\right]=0 \end{array}$$

for given values of ${\hat{ℛ}}_{0}$ corresponding to the values of

(95) $$\begin{array}{c} a_{h}=0,0.1,0.2,0.3,0.4,0.413. \end{array}$$

**Figure 9:**
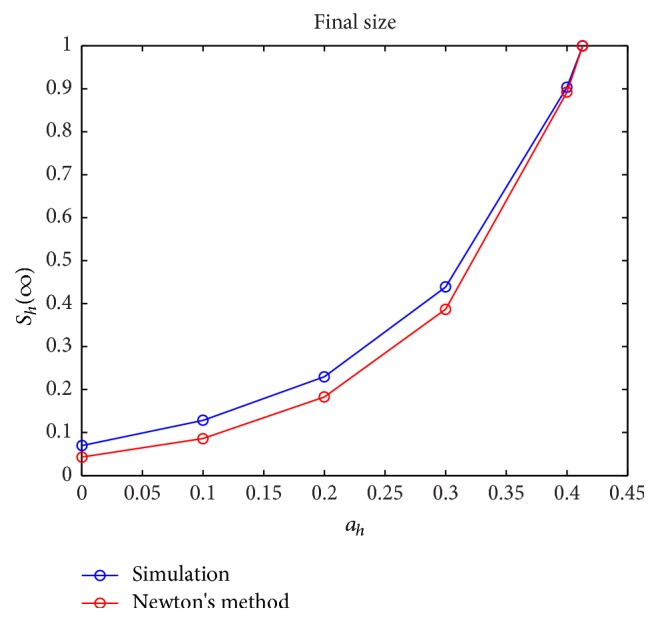
Comparison of the values of *S*_*h*_(*∞*) for ITN = 0 and increasing values of *a*_*h*_ = 0,0.1,0.2,0, 3,0.4,0.413.

Specifically, we used Newton's method for solving nonlinear equations. The comparison of the results obtained from the simulations of the dynamics of (1)–(7) with the results obtained via Newton's method is illustrated in Figure 9. Note that the *y*-axis values are normalized.

**Figure 10:**
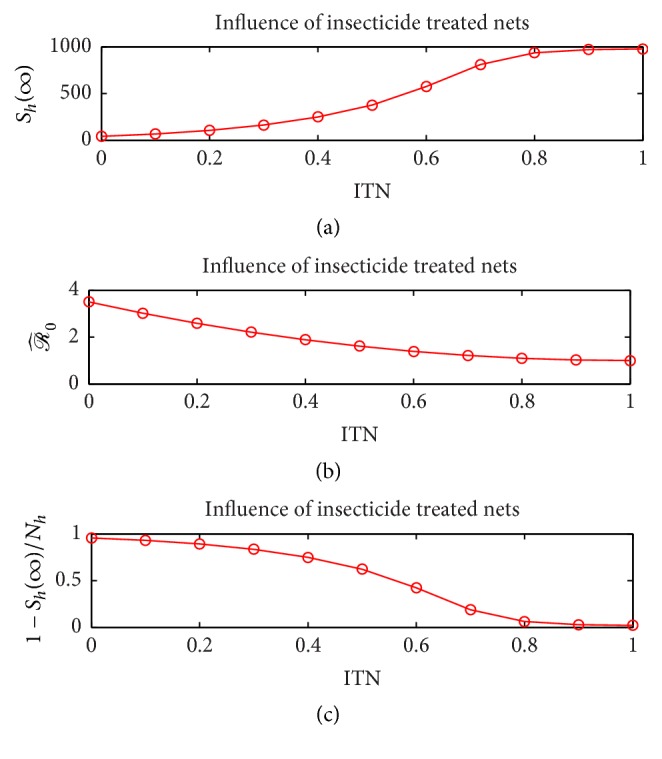
Influence of employing insecticide treated nets on final susceptible human population size (a), basic reproduction number (b), and attack rate (c).

Since it is well known that increased coverage of ITN decreases vector prevalence, we performed our next computational experiment to explore the influence of using increasing values of ITN on the dynamics. The range of values included the absence of nets (ITN = 0) to completely protective nets (ITN = 1). The results are illustrated in Figure 10 which plots the final number of susceptible humans *S*_*h*_(*∞*), the basic reproduction number ${\hat{ℛ}}_{0}$, and the attack rate 1 − *S*_*h*_(0)/*S*_*h*_(*∞*) for increasing values of ITN. The value of *a*_*h*_ was chosen to be 0.2 for this simulation that corresponds to the inclusion of both vector and direct transmission in the model. As expected, Figure 10 illustrates the usefulness of insecticide treated bed nets to control the epidemic. The figure also can potentially help government officials to decide the level of control measures through insecticide treated bed nets that is needed in a certain area to contain the spread of the epidemic.

**Figure 11:**
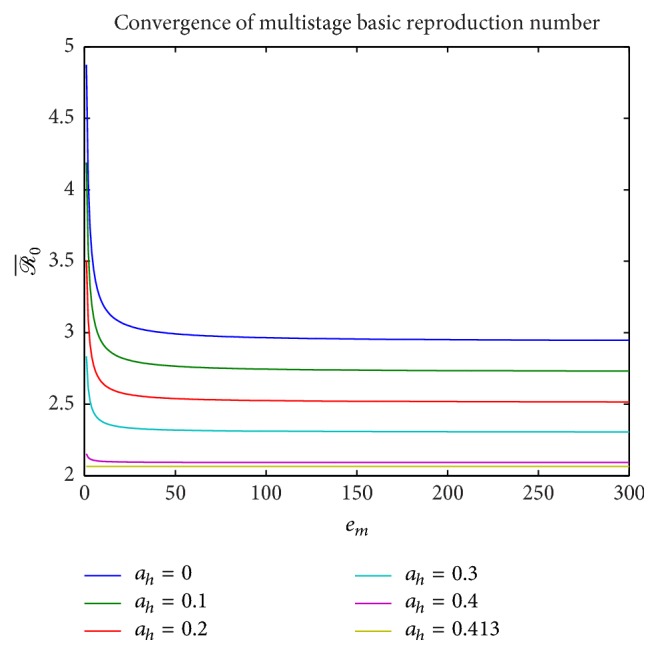
Comparison of the basic reproduction numbers for multiple incubation stages *e*_*m*_ = *e*_*h*_ = 1,…, 300 for increasing values of *a*_*h*_ = 0,0.1,0.2,0, 3,0.4,0.413 with ITN = 0.

**Figure 12:**
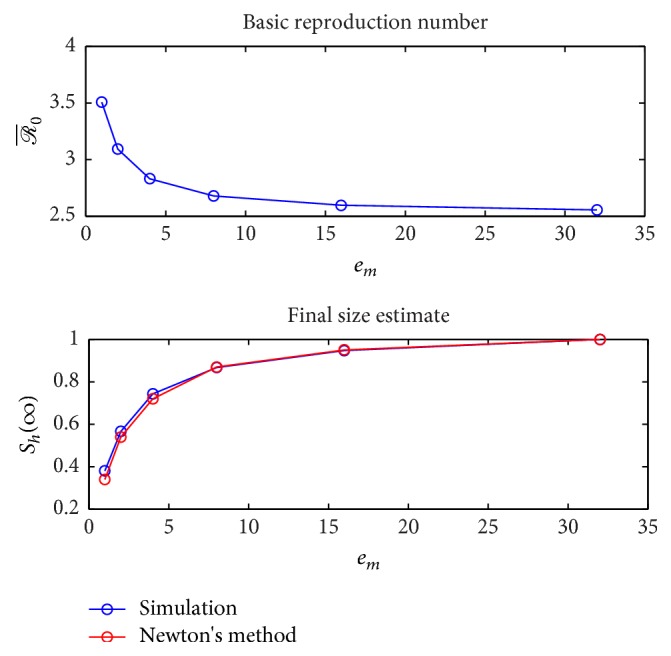
Convergence of the basic reproduction number and final size for multiple incubation stages *e*_*m*_ = *e*_*h*_ = 1,2, 4,8, 16,32 for *a*_*h*_ = 0.2 with ITN = 0.

Next, the effect of employing a multistage model on the value of the basic reproduction number ${\bar{ℛ}}_{0}$ derived in Theorem 12 is illustrated in Figure 11 for increasing values of *a*_*h*_ = 0,0.1,0.2,0.3,0.4,0.413. As the number of stage-progression increases the value of ${\bar{ℛ}}_{0}$ converges for different values of *a*_*h*_. Finally, a convergence study was performed for the full multistage system (48)–(56) for increasing number of stages doubling each time, to demonstrate that the multistage model parallels the single-stage model.  Figure 12 illustrates this convergence for fixed value of *a*_*h*_ = 0.2. Note that the *y*-axis values of the human population are normalized.

## 5. Discussion and Future Work

Over the last several decades, there has been an explosion in the development of mathematical models for outbreaks of infectious diseases that has become part of assessing epidemiological phenomena and making health policy decisions [1, 12, 27, 28]. The outbreak of any new disease has always provided both an opportunity and a challenge for mathematicians and scientists. The opportunity leads to the development of improved models and the challenge is to make sure that these models represent reality. Zika is a great example of such as disease [29]. While there is a lot of published work and information available on models, methods, and simulations on infectious diseases that are purely vector-transmitted such as malaria, Dengue, and chikungunya and diseases that spread through direct transmission only such as influenza and AIDS, new diseases such as Zika that includes both vector transmission and direct transmission has provided a challenge for new mathematical models. For example, one of the essential mathematical tools in understanding disease dynamics is the calculation of the* basic reproduction number* that can help make informed decisions on whether there will be an epidemic or not. Another important quantity is the* final size relation* that provides a useful relation between the basic reproduction number and the size of the epidemic. While there has been a lot of progress made in computing these for various types of diseases, there is still a lack of complete understanding of these quantities for vector-borne diseases such as Zika that also includes direct (sexual) transmission.

Based on the approach we take, one can obtain different measures of the basic reproduction number for vector-borne diseases. However, there are no exact analytical solutions for the final size relation for such diseases. Nevertheless, one can obtain sharp estimates with an upper and lower bound for the epidemic size [14]. The latter formulated and analyzed a model with infectivity depending on age of infection. The work provided a useful upper bound and lower bound estimate for this age of infection model that applies to vector-borne diseases such as Zika that also includes direct transmission. In this work, we provide an alternate approach to determining similar estimates for the final size relation for enhanced SEIR-SEI single-stage and multistage progression models. The multistage model for Zika considered herein includes multiple incubation substates and was motivated by similar models for Dengue that did not account for the direct (sexual) transmission. Towards this end, we are able to successfully derive a new upper bound and lower bound estimate for the final size relation for the models considered. Moreover, we are able to show that the basic reproduction number for the multistage model proposed converges to the basic reproduction number corresponding to an equivalent nonlinear system of delay differential equations with fixed incubation periods in the humans and vectors.

Another contribution in this work is the inclusion of insecticide treated nets (ITN) that offer a mix of personal protection—blocking the bites of mosquitoes, thereby reducing the transmission from mosquitoes to humans—and community protection—reducing the longevity of mosquitoes and therefore the prevalence of the infectious stage of the disease, in mosquitoes. All the results developed in this work including the basic reproduction number and the final size relation estimate incorporate the influence of the ITN which are recommended by both the CDC and WHO as effective control measures.

We hope that the models, methods, and result from this work can help provide more insight into the propagation of a disease like Zika. The work also provides some opportunities for new avenues for future research. The multistage progression model in this work only considers multiple substates for the incubation for humans and vectors. However, one may also extend this work to also incorporate multiple infectious states in humans and compare such models against standard epidemic models with fixed incubation periods in both hosts and vectors and an exponentially distributed infectious period in hosts. One may also consider including* indoor residual spraying* (IRS) that can provide a coating of the walls and other parts of a house with a residual insecticide that can kill the vectors when they come in contact. One of the assumptions in this work involves employing a removal rate *pI*_*m*_ of vectors, and we need to assume vectors are in a quasi-steady state equilibrium depending on *p* in order to continue to assume a balance relation for the host and vector contact rates. The parameter *p* indicates the dependence of the model on bednets; it could also include a rate of killing of vectors by spraying, or we might wish to include separate control parameters for the effect of bed nets and the effect of spraying. Then an interesting question would be how the basic reproduction number and the final size of the epidemic depend on these control parameters. Finally, to model the effect of bednets, one may need some terms in the host equations that could resemble those in vaccination models. For example, this can include two susceptible compartments, with a rate of transfer from susceptibles without bednets to susceptibles with bednets who would have a smaller contact rate. In this case the rate of using bednets would be a control parameter. Finally, the derivations presented herein assumed that the initial population and the steady state population of the mosquito are the same. One can relax this assumption and as pointed earlier one can rederive the estimates. While the upper bound estimate can be derived in a similar fashion as in this paper, obtaining a sharp lower bound estimate will require some work. All these features and extensions will be considered in a forthcoming paper.

## Figures and Tables

**Table 1:** Parameter definitions, values, references, and units.

Param.	Description	Value	Ref.	Units
*b*	Biting rate of vector	.5	[20]	Day^−1^
*a*_*h*_	Sexual transmission rate of Zika	0–4.13	[14]	Day^−1^
*ν*_*h*_	Human incubation rate	.14	[14]	Day^−1^
*ν*_*m*_	Vector incubation rate	.077	[14]	Day^−1^
*γ*_*h*_	Human recovery rate	.2	[14]	Day^−1^
*μ*_*m*_	Natural death rate of vector	.11	[14]	Day^−1^
*β*_*hm*_	Human to vector infection rate	.5	[21]	Day^−1^
*β*_*mh*_	Vector to human infection rate	.4	[20]	Day^−1^
*h*	Parameter for ITN rate	.003	[22]	Day^−1^
